# Observations on the Admission of Medical Pupils to the Wards of Bethlem Hospital, for the Purpose of Studying Mental Diseases

**Published:** 1842-07

**Authors:** 


ART. VI.—*Observations on the Admission of Medical Pupils to the Wards of Bethlem Hospital, for the purpose of studying Mental Diseases.* By JOHN WEBSTER, M.D. Second Edition.—*London,* 1842. 8vo, pp. 32.

THIS is a most seasonable appeal to the common sense of the Governors of Bethlem Hospital, and cannot fail to carry conviction to their minds, if they indeed possess the quality we have assumed. We think the whole profession and the public are under great obligations to Dr. Webster for his exertions in a cause incalculably important to both. No medical man, after reading the statements and arguments of the author, can fail to be astonished that such a glaring anomaly should still exist as the exclusion from the course of study prescribed for students, of one of the most important diseases in the whole range of nosology, and one which every practitioner must sooner or later meet with in his every-day business; and we think that no well-informed layman can rise from Dr. Webster's pages and fail to perceive the absolute necessity of doing away with this anomaly. Dr. Webster shows in the most satisfactory manner that so far from the visits of pupils to lunatic asylums being injurious to the inmates, they are positively beneficial to them; a fact which would seem to remove the sole objection that has any show of force against the proposition advocated by him. In the latter part of his pamphlet, Dr. Webster notices the fact of Dr. Conolly being now actually engaged at Hanwell in giving a course of gratuitous clinical lectures on insanity to pupils from the different metropolitan medical schools; and we can add, from our own knowledge, that these lectures have been already productive of results of the most beneficial kind. In the case of Dr. Conolly, we see a memorable instance of the importance it may be to society that public offices should be well filled. By acting, at last, on the "Detur Optimo" principle, the governors of Hanwell have indirectly the glory, not merely of leading the way, in this country, to remove from the medical profession the astounding imputation of ignorance of the nature and treatment of insanity, but of having taught the whole world, through the personal and literary labours of their physician, that the time has at length come when no one shall henceforth dare to treat the unhappy lunatic, of high or low degree, as a criminal, to be punished; but as a patient, to be commiserated and gently dealt with. If Dr. Webster, as we hope and believe, shall be the means of ultimately converting our metropolitan asylums into clinical schools, like that now open at Hanwell, he will have just reason to pride himself on the accomplishment of a feat which would do honour to any name in the profession. With the view of assisting him in his endeavours, we earnestly entreat all our readers not merely to peruse his pamphlet but to recommend it to the notice of all their influential friends out of the profession. Its matter cannot be better; its style may be not a little improved by the most ordinary degree of attention to the common rules of writing.

